# Documenting biodiversity with digital data: comparing and contrasting the efficacy of specimen‐based and observation‐based approaches

**DOI:** 10.1111/nph.70406

**Published:** 2025-08-07

**Authors:** Rebecca C. Wilcox, Anthony E. Baniaga, Avery P. Hill, Alison Young, Rebecca F. Johnson, Sarah J. Jacobs

**Affiliations:** ^1^ Institute for Biodiversity Science and Sustainability California Academy of Sciences San Francisco CA 94118 USA; ^2^ Department of Ecology and Evolutionary Biology, UCLA Mathias Botanical Garden University of California Los Angeles Los Angeles CA 90095 USA; ^3^ iNaturalist San Rafael CA 94915 USA

**Keywords:** California, California Floristic Province, collection bias, community science, georeference, herbarium, iNaturalist

## Abstract

- Digitized herbarium specimens and iNaturalist observations provide invaluable plant biodiversity data. Combining these two data sources could create a more holistic representation of local biodiversity; however, understanding biases inherent to each is critical to determine how to best combine and utilize these data.
- We examined how the interpretation of taxonomic and phylogenetic diversity, naturalized species detection, and spatiotemporal coverage differ when using herbarium and iNaturalist data alone and together. We also examined how these patterns vary among areas with different degrees of collecting and community science efforts.
- Across areas, diversity was higher when data sources were combined, and complementary spatiotemporal coverage between data sources indicates that combining these data is useful; however, biases unique to each data source should be considered during analyses. Naturalized species detection, diversity patterns, and spatial biases varied by area, indicating that local context impacts our current views of biodiversity and should shape future monitoring.
- Our findings suggest that continued digitization and georeferencing of the herbarium records will help provide critical information about biodiversity, but a strategic collection of both specimens and iNaturalist observations moving forward will ensure that we are capturing biodiversity change in real time, helping us track responses to environmental change.

## Introduction

Documenting and characterizing the plant diversity of an area is a long‐standing goal of naturalists and botanists. However, plant diversity is under threat from stressors associated with a rapid environmental change, and a key challenge is documenting how biodiversity is responding to those stressors (Meineke *et al*., 2018). Historically, naturalists and botanists documented biodiversity by generating herbarium specimens that cumulatively represent the biodiversity of a given area. Recent efforts to digitize and georeference current and historic specimens have been an important step in mobilizing these data (Soltis *et al*., 2018; Davis, 2023). More recently, community scientists (i.e. citizen scientists) have been able to contribute to biodiversity science through platforms such as iNaturalist (www.inaturalist.org) and have made substantial contributions to biodiversity observations around the world (Waller, 2019; Wolf *et al*., 2022; López‐Guillén *et al*., 2024). While both of these data sources independently have contributed to documenting species and biodiversity (Thornhill *et al*., 2017; Roberts *et al*., 2022), studies have also pointed out that biases inherent to these different data approaches can influence our picture of biodiversity (Meyer *et al*., 2016; Daru *et al*., 2017; Eckert *et al*., 2024). As the number of digitized and georeferenced specimens and community‐collected observations continues to increase (Soltis *et al*., 2018; Waller, 2019; López‐Guillén *et al*., 2024), we are presented with a plethora of information on historic and current species occurrence. However, in order to best leverage these data, it is critical that we understand the errors, biases, and specific strengths associated with each data source and identify what is gained by combining them. Doing so will ensure that we have the best available information to evaluate local biodiversity, and provide guidance for future data collection so that we can continue to efficiently track and evaluate changes to biodiversity (Heberling & Isaac, 2018; Soteropoulos *et al*., 2021).

While both the herbarium and iNaturalist records can be used to describe the biodiversity of a given area, there are unique benefits of and limitations to each data source. The herbarium records are physical specimens of plants, generally collected by trained botanists, with records that date back to the mid‐1500s (Stefanaki *et al*., 2019). Herbarium specimens are highly valued because the physical specimen is available for taxonomic/systematic analysis, and they provide an abundance of additional data (e.g. herbivore damage, phenology, morphometrics, ability to extract DNA, and examine microscopic characteristics; Meineke *et al*., 2018). However, specimen creation, preservation, and curation can be effort‐ and resource‐intensive. Furthermore, studies have shown that herbarium specimens may be temporally (spring and summer), spatially (collected near roads and herbaria), and even taxonomically biased (e.g. prioritization of native species) (Schmidt‐Lebuhn *et al*., 2013; Daru *et al*., 2017). At the same time, the herbarium records have also been shown to capture more taxonomic, phylogenetic, and functional diversity than iNaturalist data (Eckert *et al*., 2024). iNaturalist was started in 2008, and records consist of georeferenced observations (photographs in most cases) collected by community scientists. iNaturalist is free to use and is a useful tool for engaging with people to document and conserve plant diversity (Hardy & Hardy, 2018; Echeverria *et al*., 2021). For practitioners, iNaturalist is valued because it offers open‐access occurrence data that are quickly available and are often high volume (López‐Guillén *et al*., 2024). However, there can also be issues associated with data quality surrounding species identification and hard‐to‐distinguish duplicated records of the same individual plant (McMullin & Allen, 2022; López‐Guillén *et al*., 2024). Furthermore, records tend to be biased toward developed areas and conspicuous taxa (Di Cecco *et al*., 2021; Eckert *et al*., 2024).

While global and regional assessments of community science and herbarium data can provide important insights into regional biases (Meyer *et al*., 2016; Daru *et al*., 2017; Eckert *et al*., 2024), local assessments are critical to understanding how the local context influences sampling efforts and subsequent estimates of biodiversity (Ackerfield *et al*., 2024; Wenk *et al*., 2024). Furthermore, at local scales, biodiversity data are extremely important for updating specialized floras and understanding local changes in biodiversity through time that might be lost at coarser data resolution (Soteropoulos *et al*., 2021; Wenk *et al*., 2024). Variation in access (e.g. roads and topography), distance to herbaria, and local community science initiatives will all influence the density of records that exist in an area. In turn, this will influence the ratio of herbarium specimens to community science observations and associated biases. For example, remote areas with high levels of endemism may be targeted by professional botanists who will perform expeditions to these areas (e.g. collection hotspots; Daru *et al*., 2017), but the distance from a city and lack of roads (accessibility) could decrease the number of records from community scientists (Geldmann *et al*., 2016; Eckert *et al*., 2024). Comparatively, natural areas close to urban centers and herbaria might have active collecting, but the extent of directed community science efforts might highly influence the extent of community science records for an area (e.g. City Nature Challenge, 2024; Koedel *et al*., 2024).

In California, specimen‐collecting and observation‐based activities are abundant. In addition, the state has a hugely active botanical community (amateur and professional, alike) including the following: 36 chapters of the California Native Plant Society, CalFlora, Southern California Botanists, California Botanical Society, the Consortium of California Herbaria (CCH), and the California Natural Diversity Database. As a result, there is a huge wealth of herbarium specimens and observations that have been well‐curated (e.g. identification – both specimens and iNaturalist observations), and the local flora (summarized in the Jepson Manual) is well‐described and frequently updated (Baldwin *et al*., 2012; Jepson Flora Project, 2025). From a biodiversity perspective, California is home to a recognized global biodiversity hotspot (the California Floristic Province; Myers *et al*., 2000) and boasts over 6500 native species, subspecies, and varieties of plants, > 25% of which are endemic (Baldwin *et al*., 2012). The California flora is also highly threatened, and plants face stressors associated with habitat loss, climate change, and naturalized species (Harrison *et al*., 2024). Naturalized species, in particular, can be a threat to local plant communities (e.g. via competition with native plants) (Seabloom *et al*., 2006). Given the high level of active botanists, floristic diversity, and threats, having fast, efficient, and accurate methods to track biodiversity change (e.g. the presence of naturalized species) is both critical and possible.

To better facilitate the combined use of herbaria and iNaturalist record data, here we assess errors and biases by investigating how the interpretation of taxonomic and phylogenetic diversity, the detection of naturalized species, and spatiotemporal coverage differ between the two data sources. Furthermore, we perform this analysis across three areas with different degrees of specimen collection effort and community scientist networks to evaluate how patterns of error and bias vary based on local context. Working from the perspective of the average user (academic, land manager, conservation practitioner, etc.), we target an efficient and frequently used source of biodiversity data by pulling our data from the Global Biodiversity Information Facility (GBIF). This is followed by the summarization of data sources independently and in combination. This study will better inform practitioners how to best use these data to their advantage and will provide a set of recommendations for future data collections.

## Materials and Methods

### Study areas

**Fig. 1:**
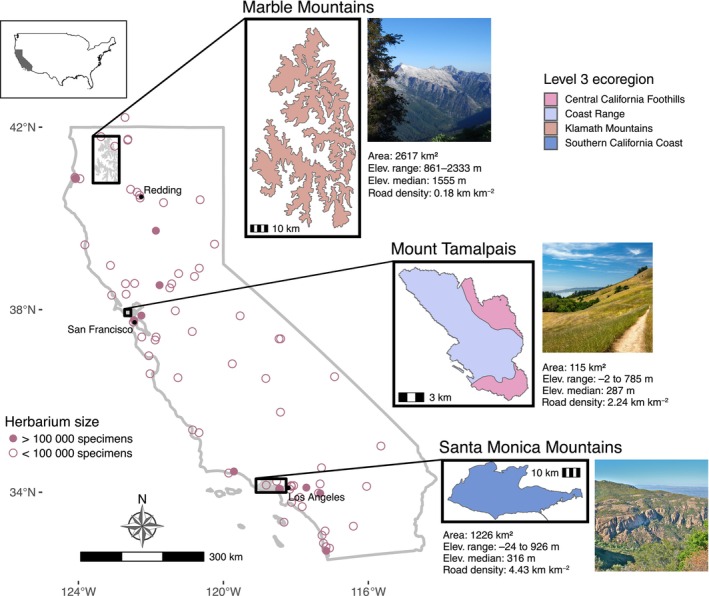
Study areas are broadly distributed across California and vary in size, elevation, and ecoregion. Herbaria include California Consortium of Herbaria contributing natural history collections and observation projects (Supporting Information Table S2). Inset maps show the exact borders of each study area and the Environmental Protection Agency Level III Ecoregion represented within (Griffith *et al*., 2016). Road density represents the density of roads within a 3‐km buffer of the study area. Sample photographs are included from within each study area (photographs from top to bottom by: Tom Hilton, CC BY 2.0; Ilya Grigorik, CC BY‐SA 3.0; Seanydelight, CC BY‐SA 3.0; see Table S3 for sources and attribution).

We chose three areas to compare for this study: the Marble Mountains, Mount Tamalpais, and the Santa Monica Mountains (SMM) (Fig. 1). These three areas are spread across California, vary in proximity to population centers, and have had differing degrees of collecting and community science/iNaturalist use. For the boundaries of these areas, we used iNaturalist ‘places’. A ‘place’ is a geographic boundary or polygon saved in the iNaturalist database that can be used as a search area for observations and a way to denote conservation statuses, common names, and checklists. We chose to use iNaturalist place polygons to define our three study areas, retrieve, and visualize data, because these are the same boundaries used to create iNaturalist projects for community science programs and events, allowing us to somewhat assess the degree of directed community science ‘effort’ in these places. For each area there were multiple polygons to choose from. Ultimately we chose to proceed with areas that had experienced different levels of development, minimized overly complicated boundaries, and maximized numbers of records (Supporting Information Table S1).

#### Marble Mountains

We used the Marble/Salmon Mountains‐Trinity Alps US EPA Level IV Ecoregion to define the Marble Mountains study area (Fig. 1), which is also a place in iNaturalist (place ID 136650). The Marble Mountains (hereafter MM) are located within the Klamath Mountains. The area is entirely made up of remote wilderness areas and is surrounded by other wilderness areas and rural towns. The plant assemblage in the MM is highly biodiverse and includes many endemic species (Sawyer & Thornburgh, 1970; Ferlatte, 1979). There are 12 herbaria within 100 km of the area, all but one of which hold fewer than 100 000 specimens (Table S2; Fig. 1). This area has seen concentrated collecting due to botanists making expeditions to the area to document its unique flora (Howell, 1944; Muth, 1973; Ferlatte, 1974). To our knowledge, there are no known community science programs or events that have taken place in the area, and only two iNaturalist projects focused on the region. The iNaturalist records from there are likely due to naturalists drawn to the area for its biodiversity.

#### Mount Tamalpais

Mount Tamalpais is the highest peak in Marin County. The majority of the mountain is contained in protected public lands, but much of the surrounding area is urban and suburban (San Francisco is *c*. 24 km south). We focused on the contiguous area of Mount Tamalpais that includes three iNaturalist places: the Marin Municipal Water District (MMWD; place ID 5500) lands, Muir Woods National Monument (place ID 5603), and Mount Tamalpais State Park (place ID 5587), all publicly accessible protected open spaces (Fig. 1). Mount Tamalpais (hereafter MT) has been a hotspot of specimen collection in the Bay Area, especially for the California Academy of Sciences (CAS) due to its close proximity. In addition to CAS, there are 10 other herbaria within 100 km of MT, three of which are moderate‐to‐large collections (collections with > 100 000 specimens; Table S2; Fig. 1). iNaturalist data in this area are also robust and growing with an active community of observers, thanks in part to community science programs such as bioblitzes and species surveys run by local organizations (e.g. OneTam). MT also hosted a unique community science project jointly run by the MMWD and CAS (2012–2017) that aimed to both collect specimens for, and document on iNaturalist, every known plant species on the MMWD lands.

#### Santa Monica Mountains

The SMMs are a coastal mountain range (part of the Transverse Mountain Ranges) of Southern California, running through southeastern Ventura and northwestern Los Angeles Counties. The range is largely encompassed by the Santa Monica Mountains National Recreation Area (SMMNRA), which not only includes many state parks and other open space preserves but also includes privately developed areas such as Malibu and the Hollywood Hills. The SMMNRA is one of the most visited natural areas in California and is considered the largest urban National Park in the world. For this study, the area of focus consisted of the SMMNRA as well as the adjacent Simi Hills (place ID 62828), a mix of publicly accessible protected open space and populated suburban areas (Fig. 1). There is a long history of plant collection in the SMMs, especially from nearby institutions such as the University of California Los Angeles (UCLA). There are 17 herbaria within 100 km of SMM, four of which are moderate‐to‐large collections (Table S2; Fig. 1). There is also a substantial dataset of iNaturalist observations from the SMMs, bolstered by community science programs and bioblitzes led by local organizations (e.g. Natural History Museum of Los Angeles County).

### Occurrence data

The initial intention of botanical specimens was to identify and document biodiversity, name new taxonomic entities, and establish type specimens, and to provide a diversity library of preserved specimens for future studies (James *et al*., 2018). Recent digitization and georeferencing of specimens have made these data more widely available and have increased novel uses of herbarium data (Soltis, 2017; Davis, 2023). Herbarium specimens are effort‐intensive, and both amateur and professional botanists will make collections. Some collectors will collect broadly, while others specialize in specific groups of plants. In California, the CCH is an organization that supports over 75 natural history collections and observation projects (here collectively referred to as herbaria) in California with resource consolidation, data aggregation, and management. CCH hosts two data portals: CCH2, which hosts and manages data from all California Herbaria, and CCH1, which aggregates data on California specimens from herbaria world‐wide.

iNaturalist is a community science platform that allows users to document biodiversity and engage in biodiversity science (iNaturalist, 2025). For a given observation, an initial identification is provided by either the observer or a combined computer vision/geomodel (or the observation is left as ‘unknown’ if no identification is added), and then corrected, refined, or confirmed by the iNaturalist community. An observation becomes Research Grade once the species identity has been agreed upon by more than two‐thirds of users providing identification on that observation. Only Research Grade observations with a Creative Commons License of CC‐BY‐NC, CC‐BY, or CC0 are sent to GBIF. iNaturalist started in 2008 and the numbers of users and observations has grown exponentially; as of April 2025, over 3.5 million people have contributed over 223 million verifiable (includes evidence, is not cultivated, and has a geolocation and date) observations to iNaturalist, 89.6 million of which are vascular plants (iNaturalist, 2025).

### Data download, data filtering, and generation of species and occurrence lists

We chose to download data from GBIF rather than directly from specific sources (CCH2, iNaturalist) for two reasons, and we acknowledge that in doing so, we make particular data concessions. First, GBIF is a global aggregator of biodiversity occurrence data, and because it has thousands of contributors and is open source, it is commonly used by practitioners seeking local biodiversity data across the world. However, occurrence data can be lost from specific contributors (data sources) because of copyright licenses (Hochmair *et al*., 2020; GBIF, 2024) or because individual contributors can vary in what data they decide to upload to GBIF (e.g. omission of locality data for rare/endangered species as protocol). Second, plant taxonomy is dynamic, and different data sources will use different taxonomic backbones to assign current species identity. For example, iNaturalist largely follows Plants of the World Online (2024) for vascular plant taxonomy at the class level and lower, with some deviations determined by the site's curators, while CCH1/CCH2 uses a Symbiota backbone curated to reflect recent updates to the Jepson eFlora. GBIF uses a multisource taxonomic backbone to unify lists that use different taxonomies and minimizes synonymy issues associated with different data sources (GBIF, 2011). This allows GBIF to group data from multiple data sources across large spatial scales; however, it can miss the most up‐to‐date local taxonomic classifications.

We downloaded all GBIF occurrences for specimen records and Research Grade iNaturalist observations of vascular plants (Tracheophyta) with geolocation data (coordinates) for all of California; then, we subset the data to include observations from each of the three areas (Hill, 2024; Derived Dataset Gbif.org, 2025). We created species lists for each of the three areas based on a unique taxon listed in the default GBIF column ‘species’. To check for errors and verify the species in each list, we examined each species for nomenclatural synonymy, geolocation error, and cultivated status. First, for each species, we searched its records for nomenclatural synonyms in the Jepson eFlora (Jepson Flora Project, 2025). Second, we examined taxonomic names to verify that an observation was congruent with that taxon's reported geographic range in California (Jepson Flora Project, 2025). We accepted a species when an observation was incongruent with the reported range as long as it was not cultivated and no element of its locality description or current species determination was obviously inaccurate. Third, we removed species from our list if they were cultivated. Occasionally, we verified herbarium specimens by finding the occurrence record in CCH2 (CCH2 Portal, 2024) or SEINet (SEINet Portal Network, 2024). We applied these filters to all species in our lists, even if they were represented only by the herbarium or iNaturalist records.

The herbarium records are often collected in duplicate, and single iNaturalist observers may take repeated observations of the same species within a close proximity. Therefore, to account for potential repeated observations, we removed duplicates of both data types using a general filter: based on geo‐location, collection date, basis of record (herbarium or iNaturalist), and collector identification before downstream summarization and analysis. We used the Jepson eFlora to determine taxonomic status in California as native or naturalized (Jepson Flora Project, 2025). All data processing and analysis steps were run in program R (R Core Team, 2024).

### Data coverage and bias

#### Taxonomic coverage and bias

To evaluate taxonomic coverage and bias, we calculated overall species richness (alpha diversity) and the number of unique species captured in each data source. For each area, we compared the taxonomic coverage between each data source using beta diversity (taxonomic turnover). We measured beta diversity with Jaccard dissimilarity (Bound 0–1), in which higher values indicate that lists are more dissimilar. We calculated beta diversity using the vegdist() function in the vegan package (Oksanen *et al*., 2024). To evaluate variation in sampling effort by species, at each area we calculated the proportion of records of each data source for each species.

#### Phylogenetic coverage and bias

To evaluate phylogenetic diversity, we used the phylogeny constructed by Thornhill *et al*. (2017), which includes all native California plants. This tree does not include plant species that are naturalized in California; therefore, our phylogenetic diversity metrics only reflect the diversity present from native species. We evaluated phylogenetic diversity using Faith's phylogenetic diversity (Faith, 1992) calculated using the pd() function in the picante package (Kembel *et al*., 2010). We assessed phylogenetic sampling bias using Pagel's λ (Bound 0–1). High values of Pagel's λ suggest sampling bias (records cluster along phylogenetic groups) and low values suggests there is little sampling bias (records are randomly distributed across the tree). We log‐transformed the number of records for each species and calculated Pagel's λ using the phylosig() function in the phytools package (Revell, 2024).

#### Temporal coverage and bias

To assess temporal bias, we examined species accumulation, changes in diversity coverage through time, and changes in sampling effort through time. We examined species accumulation by each data source to assess how the coverage of species diversity differed for the two data sources overall, and when considering native and naturalized species separately. To assess how the data sources differed in their annual coverage of taxonomic diversity through time, we examined the annual alpha diversity across time.

To determine whether the data sources differed in sampling effort through time, we examined the number of records collected and the number of unique collectors/observers each year. To evaluate the extent of fluctuations in sampling effort through time, we created two generalized additive models for each area in which response variables were as follows: the count of records per year, and the number of collectors/observers per year. We included year as a fixed effect that was allowed to vary nonlinearly (bs = ‘cr’) by data source (herbarium or iNaturalist). We did not limit the number of knots because we were interested in evaluating the extent of fluctuations through time. In both cases, we modeled the data using a negative binomial error distribution to account for overdispersion in the count data. We considered there to be a nonlinear relationship in sampling effort through time if the estimated degrees of freedom (edf) values were > 1 and the *P*‐value was < 0.05. Models were run using the gam() function in the mgcv package (Wood, 2011). We removed data from 2024 in our assessment of annual alpha diversity and our sampling effort analysis because we did not have a full 12 months of sampling for 2024.

#### Spatial coverage and bias

We analyzed spatial bias across the occurrence records by quantifying spatial autocorrelation and nearest distance to roads. We quantified spatial autocorrelation using the Nearest Neighbor Index (NNI) calculated with the nni() function in the spatialeco package (v.2.0; Evans & Murphy, 2023). This approach computes the observed Euclidean distance of each record location to its nearest neighbor and divides this by the expected distance (the average distance if points were randomly distributed). We produced NNI values for the records of each family within an area and log‐transformed them such that positive values indicate dispersion and negative values indicate clustering. We calculated NNI values for each family because sample sizes were often small at the species level. We used the Wilcoxon signed‐rank test for evaluating significant differences between NNI values of the herbarium and iNaturalist records.

We calculated the distance of the nearest road to each observation using OpenStreetMap (OSM) data and the ‘duckdb’ database engine and R package (v.1.1; Mühleisen & Raasveldt, 2024). We sourced a .pbf file of all OSM data in California from osmtoday.com on 17 November 2024 (OpenStreetMap, 2024) and loaded the file into a Duckdb database. Duckdb is a high‐performance columnar database engine that can provide dramatic computational improvements for spatial joins across large datasets. Custom SQL queries were used to find the distance to the nearest road from each point. The roads included in the dataset were tagged residential, primary, secondary, tertiary, motorway, and trunk in OSM and fell within a 3‐km buffer of the study area polygons. We included roads within a 3‐km buffer of each study area to account for roads that ended at the edge of a study area boundary (e.g. potentially leading to a trailhead or other access point). To determine whether records from the two data sources differed in their distance from roads, we used a generalized linear model for each area. In the model, distance from road was the response variable, data source was a fixed effect, and we used a Gamma error distribution (positive continuous data). Models were run using the glm() function in the stats package (R Development Core Team, 2024). From these models, we extracted parameter coefficients and 95% confidence intervals, and an effect was significant if the 95% CI around the parameter estimate did not overlap zero.

## Results

### Data download and filtering

We downloaded a total of 188 953 records from GBIF across our three areas. After filtering by nomenclatural synonyms, confirming the spatial position of geolocations, excluding cultivated occurrences, and removing duplicate records, a total of 183 078 records were retained (5875 records removed during filtering): 145 916 iNaturalist observations (80%) and 37 162 herbarium specimen records (20%) (Table S4).

Across all three datasets, a total of 2968 unique species had verifiable occurrences, which included 2158 native and 810 naturalized species. Depending on the area, between 5% and 16% of the original records were not verifiable species (Table S4). The high proportion of nonverifiable species observations in SMM (16%) is likely due to the proportionally greater amount of residential development in the study bounds corresponding to larger numbers of cultivated specimens than in other areas. Duplicate records accounted for 1.6–8.1% of records across the areas and were more common for the herbarium records (0.8–8%) than for the iNaturalist records (0.1–0.8%) (Table S5).

### Data coverage and bias

#### Taxonomic coverage and bias

The alpha diversity (species richness) of the combined datasets was 1263, 1228, and 1524 for MM, MT, and SMM, respectively (Table 1). For all areas, alpha diversity was highest when combining both lists. For MM and SMM, alpha diversity was higher when using the herbarium records compared with the iNaturalist records; the reverse was true for MT (Table 1). For MT and SMM, the iNaturalist records detected more unique naturalized species, and at all study areas, the herbarium records detected more unique native species (Table 1). Which naturalized species were most recorded varied between the two data sources, but there was some overlap (e.g. *Briza maxima* at MT and *Nicotiana glauca* at SMM) (Table S6).

**Table 1:** For each area, alpha diversity of the two data sources combined, alpha diversity based on each source separately, and the count of native and naturalized species that were unique to each data source.

Area	Alpha diversity combined	Herbarium records			iNaturalist records		
		Alpha diversity	Unique species		Alpha diversity	Unique species	
			Native	Naturalized		Native	Naturalized
Marble Mountains	1263	1194	670	58	535	57	12
Mount Tamalpais	1228	1016	148	57	1023	74	138
Santa Monica Mountains	1524	1292	256	157	1111	56	176

**Fig. 2:**
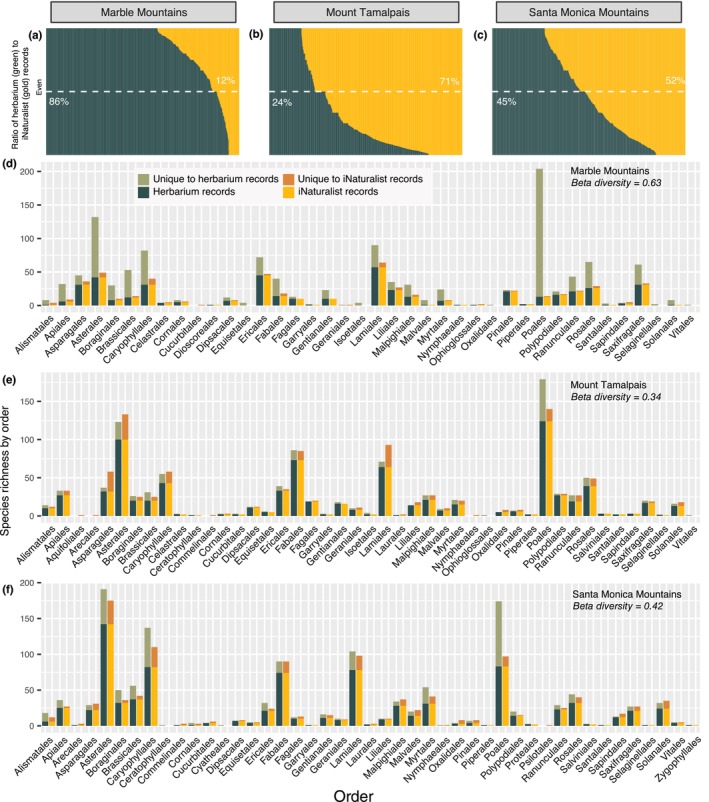
Ratio of iNaturalist observations to herbarium specimens for each species varied between the three areas (a–c). The values on the plots show the percentage of species in which the majority (> 0.5) of records were from herbarium (below the dashed line) or from iNaturalist (above the dashed line). For each order, the number of species in each list and the number of unique species to each list for (d) Marble Mountains, (e) Mount Tamalpais, and (f) Santa Monica Mountains. The beta diversity values (d–f) indicate the taxonomic turnover between lists for that area.

Seventy‐one percent of species at MT and 52% of species at SMM had more iNaturalist than herbarium records; conversely, at MM, 86% of species had more herbarium than iNaturalist records (Fig. 2a–c). Generally, the herbarium records were more likely to pick up less conspicuous species (e.g. Poales), while the iNaturalist records were more frequently associated with larger, usually abundant, conspicuous plant clades (e.g. Asterales, Lamiales; Fig. 2d–f). Taxonomically, the species lists generated by the iNaturalist and herbarium records were most similar at MT and most dissimilar in the MM (beta diversity values; Fig. 2d–f).

#### Phylogenetic coverage and bias

**Fig. 3:**
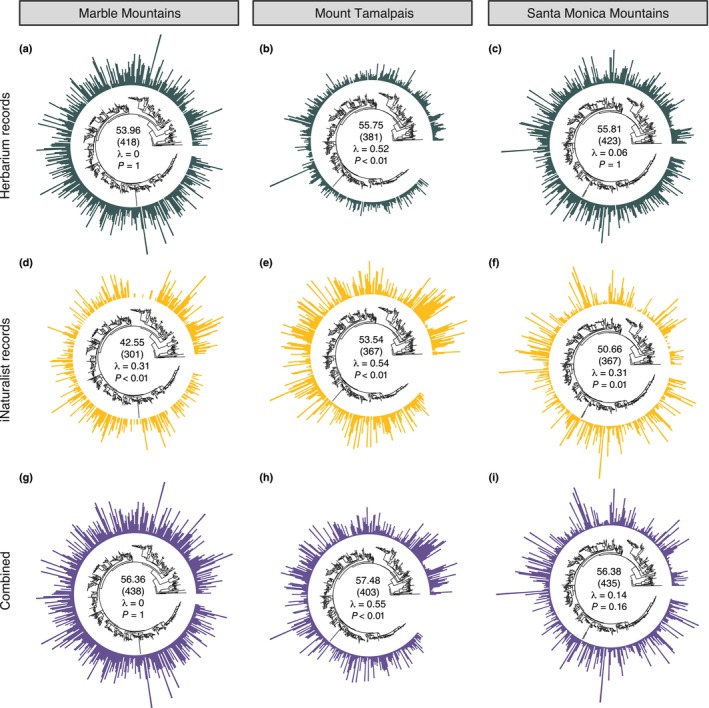
Number of records per data source (herbarium specimens, a–c; iNaturalist observations, d–f; combined, g–i) across the phylogeny of the California flora, pruned to reflect total species richness of each area. To enhance visualization, the length of the bar corresponds with the square root of the number of records. Within each tree, we report Faith's phylogenetic diversity and associated number of species (operational taxonomic units) on the tree, followed by Pagel's λ and *P*‐value.

Across areas, phylogenetic diversity was higher in the lists using the herbarium records than in the iNaturalist records and was always highest when considering both data sources combined (Fig. 3). At MM and SMM, Pagel's λ values were lower when considering the lists using the herbarium records compared with using the iNaturalist records, indicating less bias in taxonomic sampling of native species. However, for MT, Pagel's λ values were similar for the two data sources and similarly biased (Fig. 3a–c).

#### Temporal coverage and bias

**Fig. 4:**
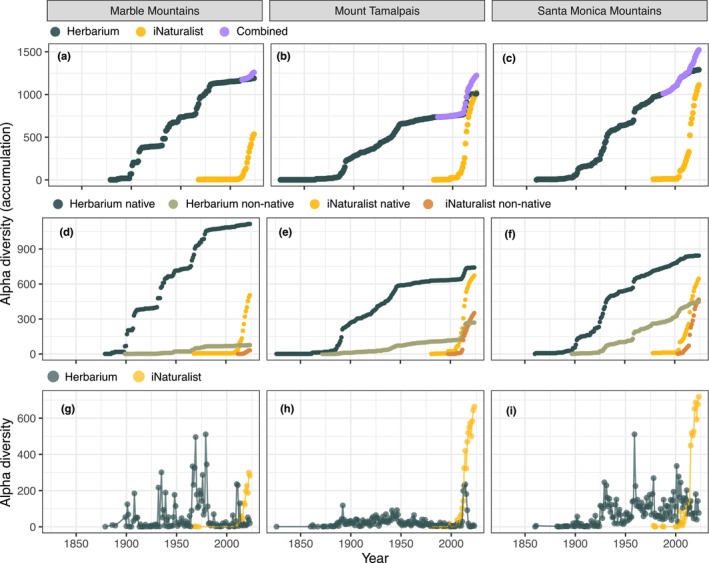
(a–c) Alpha diversity (species) accumulation for herbarium, iNaturalist, and the two data sources combined, (d–f) alpha diversity (species) accumulation of native and naturalized species for each data source, and (g–i) the annual alpha diversity sampled by each data source. The combined values (a–c, purple) start when the combined and herbarium values differ (i.e. when iNaturalist starts to add additional unique species to the area summary; Marble mountains = 2011, Mount Tamalpais = 1983, and Santa Monica Mountains = 1987).

**Fig. 5:**
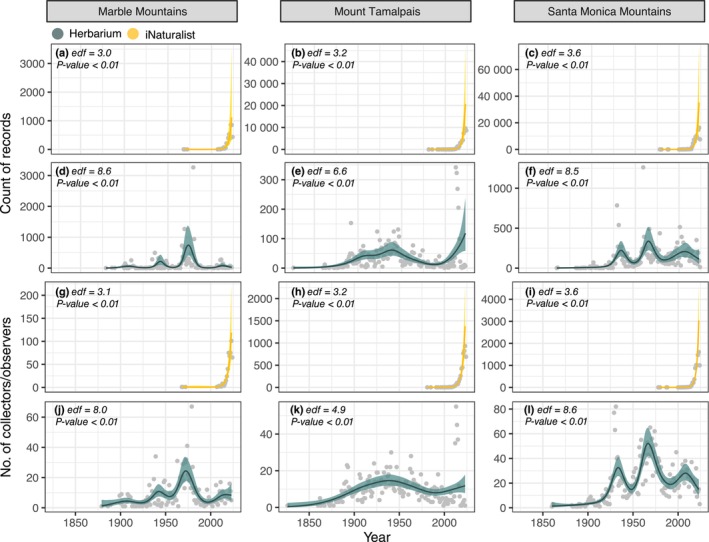
Model estimated change in the (a–f) number of records per year through time and (g–l) the number of observers per year through time for each area. The ribbons around the lines are the 95% confidence intervals, and the gray points are the raw counts. Estimated degrees of freedom (edf) values close to one indicate a linear relationship; values > 1 indicate a nonlinear relationship; the higher the value, the more nonlinear the relationship.

In all three areas, collecting for herbarium specimens began in the 1800s (1826–1879) and iNaturalist observations started in the late 1900s (1978–1981, via user uploads of photographs taken before the launch of iNaturalist in 2008) (Fig. 4). In all three areas, species accumulation is highest when considering both approaches together rather than either alone (Fig. 4a–c). At MT and SMM, iNaturalist observations have accumulated richness similar to the herbarium records in a very short period of time (20 yr), whereas at MM, iNaturalist observations have accumulated richness less than half that of the herbarium records within the last 20 yr (Fig. 4a–c). Records of naturalized species start in the late 1800s for herbarium specimens and late 1900s/early 2000s for the iNaturalist records (Fig. 4d–f). The annual alpha diversity recorded by iNaturalist has been consistently higher than herbaria since 2014 for MM, since 2013 for MT, and since 2012 for SMM (Fig. 4g–i). A similar pattern emerges when considering alpha diversity for native and naturalized species separately (Fig. S1). The number of iNaturalist records and observers far outpaced the number of the herbarium records and collectors in recent years across areas (Fig. 5). For all areas, the number of records and collectors/observers per year for herbarium was highly nonlinear compared with that of the iNaturalist records, which have consistently increased in recent decades (consistently larger and significant edf values for the herbarium records; Fig. 5).

#### Spatial coverage and bias

**Fig. 6:**
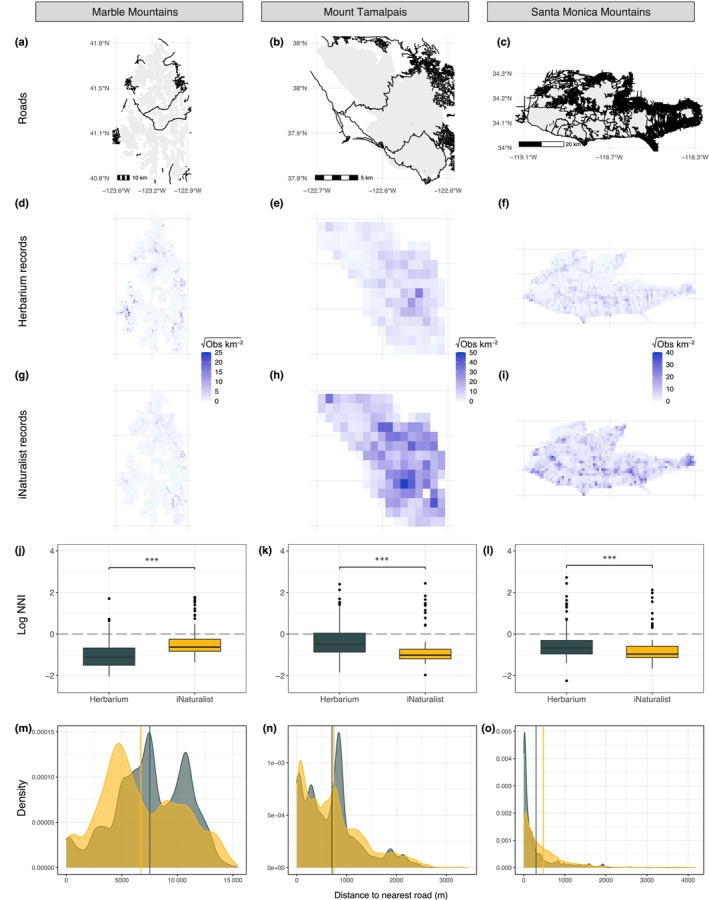
Different measures of spatial bias, including the density and dispersion of (a–c) roads within 3 km of the study area, (d–f) herbarium records, and (g–i) iNaturalist records. Nearest Neighbor Index (NNI) values were significantly different (***, *P* < 0.001; Wilcoxon signed‐rank test) between data sources across each study area (j–l). The boxplots represent the distribution of the NNI values (only statistically significant NNI values (*P* < 0.05) are shown), wherein the horizontal line represents the median, the box indicates the interquartile range, the whiskers indicate values 1.5 times the interquartile range, and the points indicate outlier values. The mean distance of each record to the nearest road was significantly different between data sources at each area (m–o). Density plots show the distribution of distance of records to the nearest road, and the vertical lines and associated ribbons show the model estimated mean and 95% confidence interval.

The data sources differed significantly in metrics of spatial autocorrelation and distance to roads. Log‐transformed NNI values were negative across all data sources and areas, indicating consistent spatial clustering (Fig. 6j–l). For MT and SMM, the iNaturalist records had significantly lower NNI values than the herbarium records (Fig. 6n,o; *P* < 0.001). Conversely, in MM, the herbarium records had significantly lower NNI values than the iNaturalist records (Fig. 6j; *P* < 0.001). The distances to the nearest road varied significantly between data sources at each area (Fig. 6m–o). SMM has the highest density of roads of the three study areas (4.43 km km^−2^ including roads within 3 km of the study area boundary; Fig. 6c), and the herbarium records were significantly closer to roads than the iNaturalist records (Fig. 6o; β = −0.46; 95% confidence interval (CI): −0.48, −0.44). Similarly, MT has a moderate density of roads (2.24 km km^−2^; Fig. 6b), and the herbarium records were also significantly closer to roads than the iNaturalist records (Fig. 6n; β = −0.05; 95% CI: −0.08, 0.03). By contrast, MM had the lowest road density of the three study areas (0.18 km km^−2^; Fig. 6a), and the herbarium records were significantly farther away from roads than the iNaturalist records (Fig. 6m; β = 0.11; 95% CI: 0.09, 0.13).

## Discussion

Documenting patterns of biodiversity is critical to our ability to protect biodiversity in the face of environmental change. Combining digitized herbarium specimens and iNaturalist observations provides an important tool to address this issue. Our results showed that given these data sources, we can summarize biodiversity patterns in a number of ways using data that are easily accessible to the average user, and when combined, these data provide a more holistic view of an area's plant diversity. There are nuances inherent to each data type which result in taxa that would be missed by relying on either source alone.

### Data coverage and bias

The herbarium records captured more taxonomic and phylogenetic diversity (with the exception of MT, in which iNaturalist observation alpha diversity slightly exceeded that of herbarium specimens). This was expected, perhaps, in MM, in which 86% of species are represented most frequently by the herbarium records, and both native and naturalized unique taxa numbers are much higher than unique iNaturalist observations. It was, however, somewhat surprising in SMM, in which only 45% of species are recorded most frequently by the herbarium records and phylogenetic diversity of herbarium specimens relative to iNaturalist observations is only *c*. 5 units higher. However, considering the high numbers of both native and naturalized unique specimen records, this finding indicates that herbarium specimens provide important information about some less observed species in SMM (e.g. members of Asterales, Caryophyllales, and Poales). At MT, we see a different pattern: only 24% of species are more frequently represented by herbarium specimens, and both specimens and iNaturalist observations have similar phylogenetic diversity estimates, and each provide large, complementary proportions of unique native and naturalized taxa (specimens and observations, respectively). This contrasting pattern among two heavily botanized areas with close proximity to large population centers is likely explained by a combined collection and observation effort centered on MT (to be described later). It is also important to note that our measures of phylogenetic diversity did not include naturalized species, which could change the patterns of phylogenetic diversity we observed.

We found that the herbarium records have a long‐standing history of recording naturalized species in all regions, indicating that these data can be used to help evaluate and assess the spread of invasive plant species (Delisle *et al*., 2003). We also found that in localities next to urban areas (MT and SMM), iNaturalist recorded more naturalized species than the herbarium records in a very short amount of time and that across sites, the iNaturalist records are recording a higher annual diversity of naturalized species. This complements literature that suggests that community scientists can be a valuable tool to track the rapid spread of introduced and invasive species (Crall *et al*., 2015; Roger *et al*., 2024).

While specimen records cover a greater temporal breadth, we found that herbarium specimen collection occurred in pulses compared with the iNaturalist records. These pulses of effort likely occur because collecting efforts are often associated with particular individuals, herbaria, or forays/projects. For example, the UCLA campus was moved to the SMM in 1929, and many of the resident UCLA faculty began actively collecting near campus, explaining the pulse of collecting pre‐1950. A second collecting pulse occurred in the 1950s–1960s and coincided with the effort to create the first published flora of the SMM (Raven & Thompson, 1966). We see similar patterns in the MM and MT. At MM, there was a 1940s pulse associated with the collecting efforts of Howell and Tracy, and a 1960s–1980s pulse associated with Muth and Ferlatte's efforts to write the flora of the Klamath Mountain Ranges. At MT, there were collection pulses in the 1900s–1940s with collecting efforts of Eastwood between 1884 and 1945 and Howell's creation of the Marin Flora (Howell, 1949). Researchers in other localities have also observed inconsistencies in specimen‐collecting effort through time, which suggests that while herbaria hold important historical records, practitioners should consider inconsistency in sampling effort when examining temporal changes in biodiversity (e.g. invasive species; Delisle *et al*., 2003).

We found variation in both spatial clustering and distance to road based on data source and area. Both the herbarium and iNaturalist records are spatially clustered, unsurprising because the collection by both methods is often opportunistic, especially with the latter. The data source with the most records in each area also had the most clustering (lowest log NNI), suggesting that as additional records are collected, they are often near the location of previous records. We expected that the herbarium records would be distributed farther from roads than the iNaturalist records, because botanists might be more likely to hike farther in search of particular species or collecting sites. This was the case at our most remote study area MM; however, the opposite was true at the other two more urban study areas, suggesting that collectors will utilize roads when available. For example, historical collecting efforts at SMM in the 1960s selectively used major roads to maximize their collection efforts. In SMM, these major roads bisect the range through valleys that exist as natural ecotones between the dominant and inaccessible chaparral slopes and other plant communities and, as such, are important access points to the flora. Additionally, SMM had the highest road density of all three areas (indicating the highest level of accessibility); the herbarium records were closer to roads than the iNaturalist records, and the herbarium records consistently recorded higher levels of diversity (phylogenetic and taxonomic) than iNaturalist data. This suggests that even with high accessibility, the iNaturalist records capture lower diversity than the herbarium records at this particular location, potentially due to the majority of observers focusing on the most noticeable or vibrant plants or a lack of targeted observation efforts. This strongly indicates that for local scales, herbarium specimens are critical for the documentation of rare and cryptic taxa, even in highly accessible and frequently visited areas. Together, our results indicate that regardless of location or data source, practitioners should consider how spatial clustering and accessibility will influence the sampling bias and taxonomic coverage when using these data.

### Pairing herbarium and iNaturalist data

In all three areas examined for this study, combining data from the iNaturalist and herbarium records produced the most comprehensive understanding of plant biodiversity; each source of information had its strengths and weaknesses, providing both redundant and complementary coverage of species in each area. While this sampling complementarity is useful, given the different strengths of the two data sources, we argue that ideally, data sources should be taxonomically redundant to maximize data complementarity. Herbarium specimens contain a valuable history of biodiversity within a region and provide a physical specimen, which allows for extensive and extended research (Meineke *et al*., 2018; Soltis *et al*., 2018; Lendemer *et al*., 2020). For example, to study naturalized species, researchers have used the genetic material from specimens to study patterns of spread and cryptic invasion (Saltonstall, 2002; Bieker & Martin, 2018; Bradshaw *et al*., 2021), which highlights the utility of having physical specimens. Compared with herbarium specimens, iNaturalist observations can be collected at a very high pace and scale, giving us a year‐over‐year understanding of biodiversity, and the photographs in iNaturalist observations also provide information such as color characteristics of living organisms and species associations (e.g. a pollinator visiting a flower), which complement the herbarium records (Heberling & Isaac, 2018). In all areas, iNaturalist data collection effort (number of collectors/observers and the number of records per year) and annual taxonomic coverage have surpassed the herbarium records in recent years, suggesting community science can be an important way to rapidly assess species occurrences even in remote areas (Crall *et al*., 2015; Soteropoulos *et al*., 2021). Therefore, a strategic collection of these specific data types can be useful, but given the complementarity of the data inherent to these two data sources (e.g. high spatial and annual temporal resolution of iNaturalist observations, opportunities associated with having a physical specimen and historic data), taxonomic redundancy between the two data sources would help ensure that practitioners have the best available data to ask a diversity of research and conservation questions. Furthermore, we emphasize the importance of future work to link the herbarium records and observation‐based records together to maximize their combined utility (e.g. James *et al*., 2018).

The area in this study with the most taxonomic redundancy was MT, likely due to the unique community science project undertaken on MMWD lands (O'Connor, 2015). The project engaged botanists and volunteers in both collecting specimens and making iNaturalist observations in an effort to photographically document and collect every plant species in the watershed. This project started by surveying plants within specific plots then evolved into an expedition‐style search with groups looking for new and unfound species. Ultimately, the project documented over 840 plant species (A. Williams *et al*., *et al*., 2017, unpublished), adding over 100 new taxa that were not on the original MMWD list and preliminarily identifying 60+ species from the original list that had likely been extirpated from these lands since the time of initial collection. This paired collecting and observation effort led to increased taxonomic redundancy and data complementarity. For example, the effort increased the number of naturalized species in herbarium, and it contributed to a lower sampling bias based on phylogeny in the iNaturalist records relative to the other two areas; taxa usually underrepresented in iNaturalist data, such as grasses, were better represented at MT. Projects like this are a valuable way to efficiently track biodiversity (with complementary data; Heberling & Isaac, 2018) and engage with the local community to promote biodiversity conservation (Soteropoulos *et al*., 2021).

### Recommendations for practitioners

The results of our study lead us to recommend that practitioners of plant biodiversity science working at local scales (e.g. researchers, conservationists, and land managers) consider three key aspects. First, all efforts should be made to maximize the representation of digital data in their initial data search. Across all three sites, we found consistent evidence that consolidating evidence from both sources (herbarium and iNaturalist) captures the maximum amount of available alpha diversity. We suggest starting with GBIF but underscore the importance of investigating the existence of other useful resources that may exist. Relevant examples for the California flora include CCH2, CalFlora, and the CNDDB.

Second, manual curation of the species list is necessary and time‐consuming, involving both taxonomic cross‐checking and occurrence validation, but is especially important at smaller (e.g. local) scales (Wenk *et al*., 2024). When data are downloaded from GBIF, the taxonomic identification from disparate sources (e.g. herbarium records and iNaturalist observations) is standardized according to a backbone taxonomy. Often, this GBIF taxonomic backbone does not include recently applied names from local taxonomic efforts. This is because, in part, these broader taxonomies rely on the monographic work that considers the entire range of a species' distribution and are typically generated at very slow rates (Davis, 2023). Such was often the case in our experience with the California flora where the Jepson eFlora treatment of vascular plants (Jepson Flora Project, 2025) tended to recognize regional varieties and subspecies more so than the broader taxonomies of GBIF, iNaturalist, and Symbiota. The taxonomic cross‐checking step took a considerable amount of time and effort, but approaches and tools to harmonize taxonomies from various sources are actively under development and will be an important tool for future users of these types of data (Grenié *et al*., 2023). Related to the taxonomic cross‐checking, the manual validation of GBIF occurrence data also took a considerable amount of time. Specifically, a small but important proportion of the species were represented only by cultivated occurrences that were erroneously listed as wild organisms. Our manual verification of these species indicated that commonly these species are naturalized in other parts of the world (but not in our local sites) and had thus become Research Grade observations on iNaturalist. We also found that this was much more frequent in SMM, which includes a significant urban and suburban population, and a place where the connection between cultivated plants and people is frequent. We recommend a data‐sorting method that uses the number of records per species as a screening tool for the species list validation step. Almost all of these types of incorrectly recorded cultivated observations of species had fewer than five records in our GBIF downloads.

Our third recommendation to practitioners is to investigate potential sources of spatial uncertainty in their digital data. This is important because although each georeferenced observation is assigned geospatial coordinates, the accuracy or uncertainty of these coordinates varies. Notably, most iNaturalist observations tend to have uncertainty values with higher precision than herbarium specimens. This is because the precise geolocations of observations in iNaturalist can be retained from a user's photo metadata with minimal user effort. By contrast, recording or estimating the georeferenced geocoordinates of herbarium specimens can require considerable manual effort and (in the case of estimation) may necessarily be associated with low precision due to the brief locality details of many historical specimens (Wenk *et al*., 2024). We found that 34% of our herbarium specimens lacked coordinate uncertainty values (Table S7) and strongly echo recommendations to add uncertainty values to georeferenced specimens (GBIF, 2024) as they can be an effective filtering criterion when present.

Additionally, we discovered that many rare, threatened, or endangered species have their precise geolocations intentionally obscured and the source of obfuscation can come from both data sources. Although herbarium specimen collectors can and do hide locality details, often the protection of locality details for sensitive species is enacted at the institutional level (e.g. a given herbarium's protocols) much before these data reach GBIF. By contrast, iNaturalist curates a list of sensitive species and increases the public positional accuracy to the diagonal of a 0.2 × 0.2 degree cell with the geolocation assigned to a random point within. Then, when these observations are sent to GBIF, GBIF shows the obscured geolocation and adds the potential obscuration distance as coordinate uncertainty. In order to account for these observations, we recommend increasing the polygon bounds of a biodiversity survey by 25 km if sensitive species are expected in your dataset, and reaching out to local experts or land managers to identify rare species for your area.

### Future directions

Our study revealed that leveraging both herbarium specimens and iNaturalist data is essential for creating the most holistic picture of local biodiversity. Moving forward, strategic and thoughtful observation campaigns, accession protocols, and collections will help ensure that future datasets maximize taxonomic, phylogenetic, temporal, and spatial coverage. One of iNaturalist's strengths is that data can be collected at high volume (particularly relative to specimens in the past decade). Therefore, deploying iNaturalist campaigns to encourage participants to search for less frequently targeted taxa, or record species in remote areas (e.g. Marble Mountains), could help to increase taxonomic and spatial coverage of occurrence records as well as increasing taxonomic redundancy with herbarium specimens in those areas (O'Connor, 2015; Soteropoulos *et al*., 2021). Furthermore, the iNaturalist platform has other features that can be useful for local practitioners – for example, users can create places that help to organize data specific to those locations – and allow users to easily examine annual biodiversity summaries to track sampling effort and data coverage. The more that practitioners engage with the iNaturalist community and provide identifications and helpful suggestions, the more this community science effort will continue to improve and develop.

Botanical collections provide a wealth of historic, contemporary, and future data in the form of physical specimens. Recent digitization and georeferencing efforts have provided a renaissance for the use of herbarium data (Davis, 2023; Eckert *et al*., 2024; Ramirez‐Parada *et al*., 2024). However, across herbaria, there remains a backlog of specimens awaiting digitization and, especially, georeferencing. For example, as of April 2025, of 606 921 California records at CAS, only 18.5% have been georeferenced. Therefore, not only continuing to digitize and georeference backlogs is critical for making historic data available, but also having standardized accession protocols that incorporate digitizing and georeferencing will be critical to ensure that backlogs do not reemerge and that data continue to be available. Regional efforts such as CCH or the Mid‐Atlantic Herbaria Consortium are examples of groups doing important work to digitize and curate local floras by aggregating small‐to‐large natural history collections and observation projects. Having these data available will allow herbaria to conduct and publish gap analyses in order to identify how to strategically grow individual collections and collections generally to maximize coverage and minimize bias. We should also ensure that herbaria are well‐funded, staffed, and resourced and that universities and botanical communities are still producing taxonomists/botanists sufficient for the task of collecting specimens and curating our taxonomy and classification system (Bizecki Robson & Krindle, 2025). Additionally, research shows that nuances among collectors (e.g. prolific vs less‐prolific collectors) can influence collection bias (Schmidt *et al*., 2025), suggesting that having diverse collectors ensures broader spatial, temporal, and taxonomic coverage. Therefore, increasing diversity of collectors will not only help to cultivate a robust community of collectors but also ensure ample data coverage.

All paths forward to meet our conservation challenges rely heavily on the continued documentation of biodiversity. Engaging more people in collecting these data can be an important step in instilling a sense of belonging and inclusion into the broader and long‐term conservation effort. Obtaining broad and active participation in biodiversity observations from professionals, amateurs, and community scientists alike is essential to meeting our conservation goals.

## Competing interests

None declared.

## Author contributions

SJJ and AEB conceptualized the manuscript. RCW, AEB and APH performed data curation. SJJ, RCW and APH did formal analysis and visualizations. SJJ and RFJ acquired funding. SJJ and RCW conducted project administration. SJJ supervised the project. AEB and APH performed validation. SJJ, AEB, RCW, APH and AY prepared the original draft of the manuscript. All authors contributed to the investigation, methodology, and reviewing and editing the manuscript.

## Disclaimer

The New Phytologist Foundation remains neutral with regard to jurisdictional claims in maps and in any institutional affiliations.

## Supporting information


**Fig. S1** Annual alpha diversity sampled by each data source for native and naturalized species.
**Table S1** Links to the polygons we used to designate our study areas.
**Table S2** Proximity of Consortium of California Herbaria: Natural History Collections and Observation Projects, in proximity to each of the three study areas.
**Table S3** Source information for images used in Fig. 1.
**Table S4** For each study area, a summary of the number of species and records for each data source before and after filtering.
**Table S5** For each study area, the number of duplicate records removed for each data source combined and separately.
**Table S6** For each study area, the top five most frequently observed naturalized species by each data source.
**Table S7** For each study area, after data filtering, the number of records with no coordinate uncertainty values associated with them and records with uncertainty > 25 km.
Please note: Wiley is not responsible for the content or functionality of any Supporting Information supplied by the authors. Any queries (other than missing material) should be directed to the *New Phytologist* Central Office.

## Data Availability

The data that support the findings of this study are openly available in Zenodo at doi: 10.5281/zenodo.15265921. The derived dataset from GBIF can be found here: https://www.gbif.org/derivedDataset/10.15468/dd.caemmf. Code for data access and the spatial analyses and visualization can be found at the following GitHub repository: https://github.com/avephill/documenting‐biodiversity‐with‐digital‐data.
