# Supplementary material for: Documenting biodiversity with digital data: comparing and contrasting the efficacy of specimen‐based and observation‐based approaches
Source: New Phytol. 2025 Aug 7;251(2):721–36. doi: 10.1111/nph.70406 (PMC13278658; doi:10.1111/nph.70406)
Supplement: Supplementary file 1 — Fig. S1 Annual alpha diversity sampled by each data source for native and naturalized species. Table S1 Links to the polygons we used to designate our study areas. Table S2 Proximity of Consortium of California Herbaria: Natural History Collections and Observation Projects, in proximity to each of the three study areas. Table S3 Source information for images used in Fig. 1. Table S4 For each study area, a summary of the number of species and records for each data source before and after filtering. Table S5 For each study area, the number of duplicate records removed for each data source combined and separately. Table S6 For each study area, the top five most frequently observed naturalized species by each data source. Table S7 For each study area, after data filtering, the number of records with no coordinate uncertainty values associated with them and records with uncertainty > 25 km. Please note: Wiley is not responsible for the content or functionality of any Supporting Information supplied by the authors. Any queries (other than missing material) should be directed to the New Phytologist Central Office. [file NPH-251-721-s001.docx]

## *New Phytologist* Supporting Information

Article title: Documenting biodiversity with digital data: comparing and contrasting the efficacy of specimen-based and observation-based approaches

Authors: Rebecca C. Wilcox, Anthony E. Baniaga, Avery P. Hill, Alison Young, Rebecca F. Johnson, Sarah J. Jacobs

Article acceptance date: 26 June 2025

The following Supporting Information is available for this article:

**Fig. S1** The annual alpha diversity sampled by each data source for native and naturalized species.

**Table S1** Links to the polygons we used to designate our study areas.

**Table S2** Proximity of Consortium of California Herbaria: Natural History Collections and Observation Projects, in proximity to each of the three study areas.

**Table S3** Source information for images used in Fig. 1.

**Table S4** For each study area, summary of the number of species and records for each data source before and after filtering.

**Table S5** For each study area, the number of duplicate records removed for each data source combined, and separately.

**Table S6** For each study area, the top five most frequently observed naturalized species by each data source.

**Table S7** For each study area, after data filtering, the number of records with no coordinate uncertainty values associated with them and records with uncertainty greater than 25 km.

**Fig. S1** The annual alpha diversity sampled by each data source for (a-c) native and (d-f) naturalized species. These figures do not include data from 2024.


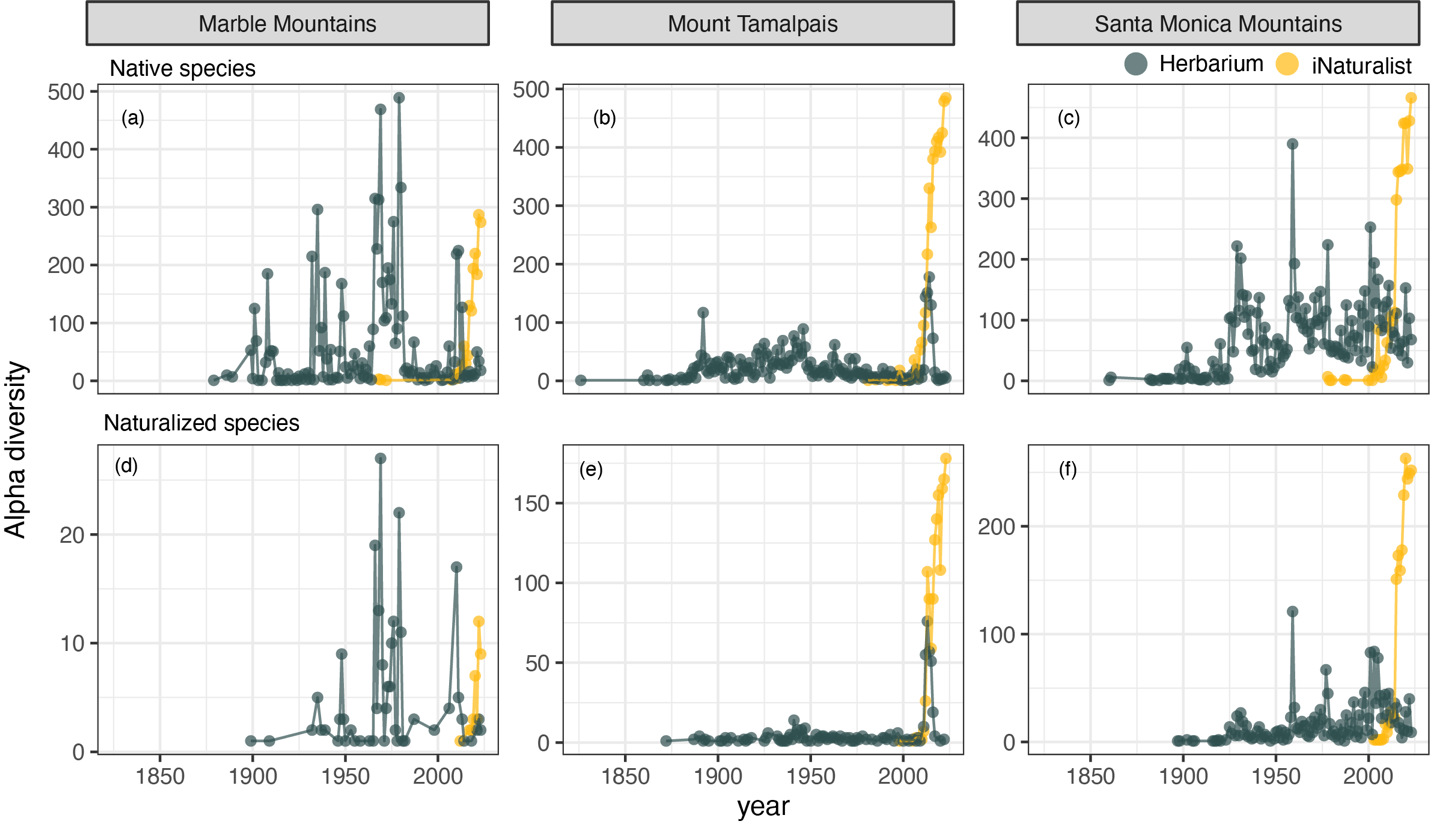


**Table S1** Links to the polygons we used to designate our study areas. For Mount Tamalpais we combined polygons from three iNaturalist places. We were interested in the contiguous areas represented by these three places therefore we removed the northern isolated property in the Marin Municipal Watershed District from our final polygon.

| Study Area | Place Name | Place ID | Place Link |
| --- | --- | --- | --- |
| Marble Mountains | Marble/Salmon Mountains-Trinity Alps* | 136650 | <https://www.inaturalist.org/observations?place_id=136650> |
| Mount Tamalpais | Marin Municipal Water District Watershed | 5500 | <https://www.inaturalist.org/observations?place_id=5500> |
|  | Muir Woods National Monument | 5603 | <https://www.inaturalist.org/observations?place_id=5603> |
|  | Mount Tamalpais State Park | 5587 | <https://www.inaturalist.org/observations?place_id=5587> |
| Santa Monica Mountains | Santa Monica Mountains | 62828 | <https://www.inaturalist.org/observations?place_id=62828> |

*Same as the US EPA Level IV Ecoregion.

**Table S2** Proximity of Consortium of California Herbaria: Natural History Collections and Observation Projects, in proximity to each of the three study areas (Fig. 1) (acronyms in parenthesis). Proximity is defined as the closest possible point of the study area to the institution. We included all California based institutions listed on the CCH2 website, and one Oregon herbarium which was in close proximity to the Marble Mountains study area (https://www.cch2.org/portal/collections/misc/collprofiles.php). Bolded herbaria indicate moderate to large collections (greater than 100,000 specimens).

| **Santa Monica Mountains** | **Mount Tamalpais** | **Marble Mountains** |
| --- | --- | --- |
| **within 10 km** | | |
| **Herbarium - University of California, Los Angeles (LA)** |  | Klamath National Forest Herbarium (KNFY) |
| Herbarium - California State University, Northridge (SFV) |  |  |
| Santa Monica & Simi Hills herbarium of the National Park service (SMMSH) |  |  |
| **within 25km** | | |
| Herbarium - California State University, Los Angeles (CSLA) | **California Academy of Sciences (CAS)** | Klamath National Forest Happy Camp/Oak Knoll Herbarium (KNFHC) |
| Huntington Botanical Gardens Herbarium (HNT) | Harry D. Thiers Herbarium, San Francisco State University (SFSU) |  |
| Los Angeles County Arboretum & Botanic Garden Herbarium (LASCA) |  |  |
| Pasadena City College herbarium (PASA) |  |  |
| **within 100km** | | |
| Catalina Island Conservancy (CATA) | The Peter G. Connors Herbarium at Bodega Marine Reserve (BMR) | Siskiyou County Dept. of Agriculture Herbarium (SCDA) |
| Riverside Metropolitan Museum, Clark Herbarium (CLARK) | UC Berkeley, Blue Oak Ranch Reserve (BORR) | Southern Oregon University Herbarium (SOC) |
| California State University San Bernardino (SCUSB) | **Herbarium - University of California, Davis (DAV)** | Sierra Pacific Industries-Forestry (SPIF) |
| Herbarium - University of California, Irvine (IRVC) | Jasper Ridge Biological Preserve, Stanford University (JROH) | Shasta-Trinity National Forest Herbarium (STNF) |
| Herbarium - California State University, Long Beach (LOB) | Sonoma State University (NCC) | Whiskeytown National Recreation Area Herbarium (WHIS) |
| MacFadden Herbarium, California State University Fullerton (MACF) | Stephen J. Barnhart Herbarium at Pepperwood Preserve (PPWD) | Klamath National Forest Scott-Salmon District (KNFSC) |
| **California Botanic Garden Herbarium (RSA)** | Pacific Union College (PUA) | BLM Arcata Field Office Herbarium (BLMAR) |
| **Herbarium - Santa Barbara Botanic Garden, California (SBBG)** | Herbarium - San Jose State University, California (SJSU) | BLM Herbarium - Redding FO (BLMRD) |
| **Herbarium - University of California, Riverside (UCR)** | **UC Berkeley and Jepson Herbarium (UCJEPS)** | Green Diamond Resource Co (GDRC) |
| Herbarium - Cheadle Center for Biodiversity and Ecological Restoration, University of California Santa Barbara (UCSB) |  | **Humboldt State University Herbarium, Arcata, California (HSC)** |
| **Total herbarium within 100km:** | | |
| 17 | 11 | 12 |

**Table S3** Source information for images used in Fig. 1 including, for photo: attribution to the image’s author, title, link to the file, license notice, link to the license, and notes on how the image was modified.

| Author | Title | Link to file | License notice | License link | Changes made to image |
| --- | --- | --- | --- | --- | --- |
| Tom Hilton | Trinity Alps Wilderness with Pinus balfouriana | https://commons.wikimedia.org/wiki/File:Trinity_Alps_Wilderness_with_Pinus_balfouriana.jpg | CC BY 2.0 | https://creativecommons.org/licenses/by/2.0/deed.en | Image was cropped |
| Ilya Griogorik | Matt Davis Trail Mt Tamalpais | https://commons.wikimedia.org/wiki/File:Matt_Davis_Trail_Mt_Tamalpais_(159382381).jpeg | CC BY-SA 3.0 | https://creativecommons.org/licenses/by-sa/3.0/deed.en | Image was cropped |
| Seanydelight | Backbone Trail near Mishe Mokwa | https://en.m.wikipedia.org/wiki/File:Backbone_Trail_near_Mishe_Mokwa.jpg | CC BY-SA 3.0 | https://creativecommons.org/licenses/by-sa/3.0/deed.en | Image was cropped |

**Table S4** For each study area, summary of the number of (a) species and (b) records for each data source before and after filtering.

|  | Herbarium | | iNaturalist | | Combined | |
| --- | --- | --- | --- | --- | --- | --- |
| Study area | Pre-filtering | Post-filtering | Pre-filtering | Post-filtering | Pre-filtering | Post-filtering |
| (a) Species |  |  |  |  |  |  |
| Marble Mountains | 1263 | 1194 | 544 | 535 | 1334 | 1263 |
| Mount Tamalpais | 1074 | 1016 | 1056 | 1023 | 1302 | 1228 |
| Santa Monica Mountains | 1494 | 1292 | 1214 | 1111 | 1809 | 1524 |
| (b) Records |  |  |  |  |  |  |
| Marble Mountains | 16046 | 14100 | 4169 | 4125 | 20215 | 18225 |
| Mount Tamalpais | 6277 | 5595 | 54368 | 53649 | 60645 | 59244 |
| Santa Monica Mountains | 19168 | 17467 | 88925 | 88142 | 108093 | 105609 |

**Table S5** For each study area, the number of duplicate records removed for each data source combined, and separately.

|  | Total number duplicate records removed (% of total records) | | |
| --- | --- | --- | --- |
| Study area | Combined | Herbarium | iNaturalist |
| Marble Mountains | 1604 (8.1%) | 1589 (8%) | 15 (0.1%) |
| Mount Tamalpais | 968 (1.6%) | 511 (0.8%) | 457 (0.8%) |
| Santa Monica Mountains | 1762 (1.6%) | 1297 (1.2%) | 465 (0.4%) |

**Table S6** For each study area, the top five most frequently observed naturalized species (n=number of records) by each data source.

|  | Herbarium | | |  | iNaturalist | | |
| --- | --- | --- | --- | --- | --- | --- | --- |
| Study area | rank | species | n |  | rank | species | n |
| Marble Mountains | 1 | *Rumex acetosella* | 41 |  | 1 | *Verbascum thapsus* | 6 |
|  | 2 | *Glyceria elata* | 33 |  | 2 | *Tragopogon dubius* | 4 |
|  | 3 | *Poa pratensis* | 28 |  | 3 | *Cirsium vulgare* | 3 |
|  | 4 | *Spergularia rubra* | 15 |  | 3 | *Digitalis purpurea* | 3 |
|  | 4 | *Trifolium repens* | 15 |  | 3 | *Phleum pratense* | 3 |
| Mount Tamalpais | 1 | *Bromus carinatus* | 19 |  | 1 | *Myosotis latifolia* | 262 |
|  | 2 | *Briza maxima* | 11 |  | 2 | *Genista monspessulana* | 193 |
|  | 2 | *Bromus hordeaceus* | 11 |  | 3 | *Briza maxima* | 179 |
|  | 2 | *Cynosurus echinatus* | 11 |  | 4 | *Lathyrus latifolius* | 126 |
|  | 5 | *Bromus madritensis* | 8 |  | 5 | *Euphorbia oblongata* | 121 |
|  | 5 | *Holcus lanatus* | 8 |  |  |  |  |
| Santa Monica Mountains | 1 | *Bromus rubens* | 43 |  | 1 | *Ricinus communis* | 882 |
|  | 2 | *Centaurea melitensis* | 41 |  | 2 | *Nicotiana glauca* | 844 |
|  | 2 | *Nicotiana glauca* | 41 |  | 3 | *Marrubium vulgare* | 829 |
|  | 4 | *Lamarckia aurea* | 37 |  | 4 | *Erodium cicutarium* | 555 |
|  | 5 | *Marrubium vulgare* | 32 |  | 5 | *Cenchrus setaceus* | 515 |

**Table S7** For each study area, after data filtering (including only verified species and removing duplicates), the number of records (and percentages) with no coordinate uncertainty values associated with them (NA) and records with uncertainty greater than 25 km (most likely associated with rare or endangered species).

|  | Records with no coordinate uncertainty | | uncertainty > 25 km | |
| --- | --- | --- | --- | --- |
| Study area | Herbarium | iNaturalist | Herbarium | iNaturalist |
| Marble Mountains | 6018 | 1118 | 6 | 644 |
|  | 43% | 27% | 0% | 16% |
| Mount Tamalpais | 1670 | 14886 | 0 | 857 |
|  | 30% | 28% | 0% | 2% |
| Santa Monica Mountains | 4992 | 17398 | 36 | 2289 |
|  | 29% | 20% | 0% | 3% |
| Total: | 12680 | 33402 | 42 | 3790 |
|  | 34% | 23% | 0% | 3% |
| Grand total: | 46082 | | 3832 | |
|  | 25% | | 2% | |
